# Type II Aortopulmonary Window with Isolated Left Subclavian Artery from Left Pulmonary Artery

**DOI:** 10.21470/1678-9741-2018-0417

**Published:** 2020

**Authors:** Vishal Agrawal, Abdul Majid, Imelda Jain, Megha Sheth, Amit Mishra

**Affiliations:** 1 Department of Cardiovascular and Thoracic Surgery, UNMICRC – U. N. Mehta Institute of Cardiology & Research Centre, Gujarat, India.; 2 Department of Cardiac Anaesthesia, UNMICRC – U. N. Mehta Institute of Cardiology & Research Centre, Gujarat, India.; 3 Department of Radiology, UNMICRC – U. N. Mehta Institute of Cardiology & Research Centre, Gujarat, India.; 4 Department of Pediatric Cardiovascular and Thoracic Surgery UNMICRC – U. N. Mehta Institute of Cardiology & Research Centre, Gujarat, India.

**Keywords:** Subclavian Artery, Congenital heart disease, CHD

## Abstract

Type II Aortopulmonary window (APW) accounts for only 10% of total cases of APW, which by itself is a rare congenital anomaly. Various cardiac malformations have been reported to be associated with this rare anomaly. We report one such association of origin of left subclavian artery (LSCA) from left pulmonary artery (LPA) via ductus arteriosus that was surgically repaired.

**Table:** 

Abbreviations, acronyms & symbols	
APW	= Aortopulmonary window
CT	= Computed tomography
CHD	= Congenital heart disease
LPA	= Left pulmonary artery
LSCA	= Left subclavian artery
PA	= Pulmonary artery
PDA	= Patent ductus arteriosus
PVR	= Pulmonary vascular resistance
SSS	= subclavian steal syndrome
VA	= Vertebral artery

## INTRODUCTION

Isolation of the subclavian artery is the loss of continuity between one subclavian artery and the aorta, with persistent connection to the homolateral pulmonary artery through the patent (PDA) or nonpatent ductus arteriosus. It is a rare anomaly mostly reported with right aortic arch^[1]^.  Leutmer et al.^[1]^described its most common association with Tetralogy of Fallot, besides various other intracardiac lesions. Aortopulmonary window is relatively a rare cardiac lesion, most commonly associated with malformations like type A interruption of aortic arch, coarctation of the aorta, Tetralogy of Fallot and aortic origin of right pulmonary artery. Zhu et al.^[2]^ has described previously an association of Type I aortopulmonary window (APW) with isolated left subclavian artery (LSCA). We here present a case of Type II APW with isolated LSCA in an infant whose connection to the homolateral pulmonary artery was atretic.

## CASE REPORT

**Fig. 1:**
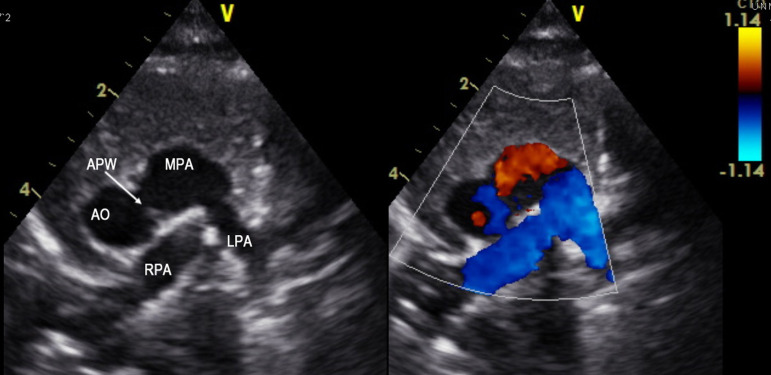
2D echocardiography images showing distal variety of aortopulmonary window with predominant left to right shunt.

**Fig. 2:**
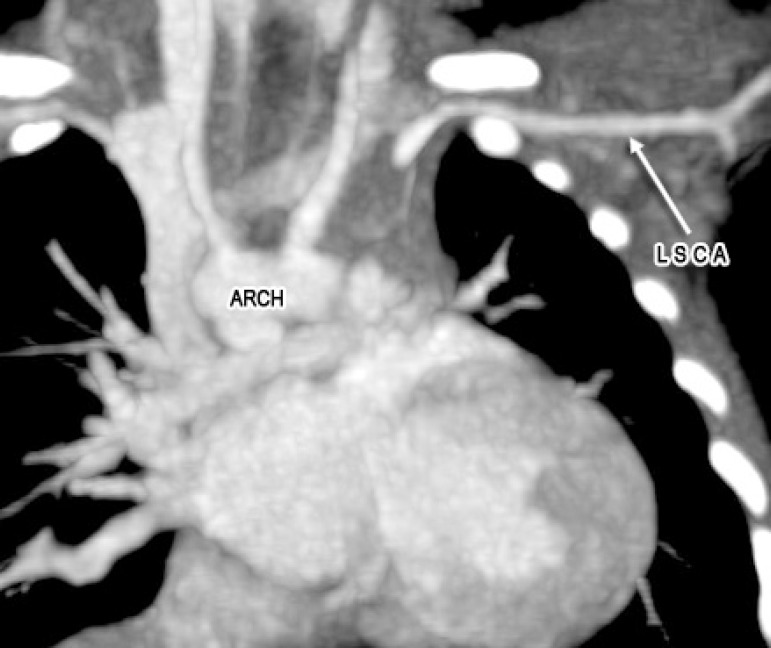
CT scan images showing isolated left subclavian artery arising from left pulmonary artery with thrombosed ductal tissue intervening them and filling by collaterals.

**Fig. 3:**
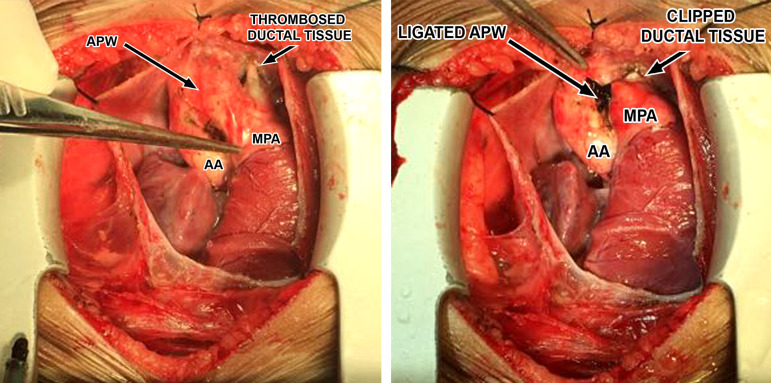
Intraoperative image showing aortopulmonary window and pulmonary artery with thrombosed intervening ductal tissue.

A 2-month old child was admitted to our hospital diagnosed with recurrent upper respiratory tract infections and feeding difficulty from birth. The infant was noncyanotic with holosystolic murmur at pulmonary area. After echocardiography, the diagnosis was right aortic arch with distal variety of aortopulmonary window (APW) of 10mm in diameter, with predominantly left-to-right shunt (Figure 1), mild mitral regurgitation and systemic pulmonary artery (PA) pressures. There were no other associated intracardiac defects. In order to delineate the anatomy better a computed tomography (CT) angiogram was done. CT angiogram confirmed the diagnosis of distal variety of APW just proximal to pulmonary artery bifurcation. There was associated anomalous origin of left subclavian artery (LSCA) from left pulmonary artery (LPA) via ductus arteriosus (Figure 2). However, the ductal connection was fully thrombosed and left subclavian artery was filling well by collaterals from left vertebral artery. Patient was re-examined and there was neither any radio-radial delay nor any pulse volume and blood pressure difference between right and left hands.  Surgical correction of aortopulmonary window was planned through median sternotomy. During the surgery, distal variety of APW was confirmed approximately 3cm above the aortic valve (Figure 3). The Ductal tissue was thrombosed until it connected to LSCA but, to be on the safer side, its connection to LPA was clipped. The APW was looped and ligated under controlled hypotension.  At the end of the operation, arterial pressure was 90/50 mmHg. The postoperative course was uneventful with extubation on the second day after the operation. The postoperative blood pressure in the left and right arm was 85/50 and 90/55 mmHg, respectively. The patient was successfully discharged in good clinical condition on the 7th day after the operation.

## DISCUSSION

Embryologically abnormal separation by conotruncal septum of truncus arteriosus results in aortopulmonary window (APW). Most of the cases are associated with various other congenital anomalies. Isolated LSCA is the loss of connection of the LSCA from the aortic arch; it originates from the homolateral pulmonary artery through a patent or closed ductus arteriosus. It is the least common variant of right aortic arch, accounting for only 0.8% of the cases^[3]^. Edward’s “hypothetical double aortic arch plan” explains the embryogenesis of isolated left subclavian artery^[4]^. There is an interruption of the aortic arch at two levels, one between the left common carotid artery and the LSCA, and the second between the left ductus arteriosus and the aortic root. This results in a right aortic arch with isolated LSCA. The branches of the right aortic arch are in order of the left common carotid, the right common carotid, and the right subclavian arteries. The LSCA becomes detached from the aorta and is connected to the pulmonary artery by the ductus arteriosus either closed or open. Di George syndrome has a very high frequency of right aortic arch anomalies, but we don’t have facilities for genetic testing in our institute.

Patients with isolated LSCA often present with symptoms of subclavian steal syndrome later in life. Subclavian ‘steal’ is a phenomenon in which the subclavian artery is filled with retrograde blood flow from the ipsilateral vertebral artery (VA). The symptoms from the compromised vertebrobasilar and brachial blood flows constitute the subcla­vian steal syndrome (SSS), and include paroxysmal vertigo, drop attacks and/or arm claudication. On examination, patients have weakened pulses of the ipsilateral upper limb and difference in blood pressure of both the upper limbs. Recent studies have shown a linear correlation between in­creasing arm blood pressure difference with the occurrence of symptoms^[5]^. For isolated subclavian from the pulmonary artery (PA), the flow could be from the PA if the proximal ductal portion was patent and, in severe cases, lead to pulmonary steal phenomenon. “Pulmonary steal” is essentially exaggerated subclavian steal whereby blood is stolen from the head via the ipsilateral vertebral, but flows retrograde down the subclavian and into the PA. The amount of flow can exceed that of subclavian steal because PVR can be considerably lower. Thus, on suspicion of symptoms of vertebrobasilar insufficiency and presence of right aortic arch in chest x-ray, the diagnosis of isolated LSCA should be looked for in 2D echocardiography or CT angiogram. In our case, there was an associated aortopulmonary window with systemic PA pressures so chances of pulmonary steal phenomenon were less. Also, the left pulmonary artery connection was atretic, so the isolated LSCA was filling retrogradely from the left vertebral artery. Hence there is always a probability that the child may develop Subclavian steal syndrome later on^[6]^. Since the ductal tissue seemed obliterated and left subclavian was filling with good collateral flow and there was no major difference in blood pressure of both upper limbs, it was decided not to reimplant the left subclavian artery in this 2-month old child. We, however, have clipped the pulmonary end of ductus to be sure to avoid pulmonary steal phenomenon.

Standard technique for a large APW with sever pulmonary hypertension is patching of APW with or without separating the great vessels using cardiopulmonary bypass. Since the APW was around 30mm above the aortic valve, just proximal to bifurcation, it was looped and ligated under controlled hypotension. Ligation always carries a risk for bleeding, and it is always safer to approach by sternotomy. Moreover, there is also a risk of recannulation of ligated APW. Considering that we were able to avoid cardiopulmonary bypass, which was less invasive for the patient, and perform ligation securely, we opted for this approach. 

On  the 6-month postoperative follow up there was no history of syncope or giddiness. On examination, there was no arm length discrepancy and no significant difference in blood pressure of both upper limbs. However, we do agree that a longer follow up will be needed to see if the patient develops features of arm length discrepancy and subclavian steal syndrome later in life.

## CONCLUSION

Isolation of LSCA in type II APW is a very rare entity. As such, isolation of LSCA doesn’t alter the prognosis of the patient but there always remains a dilemma regarding the management of isolated LSCA. We had undertaken the conservative route and did not reimplant the LSCA to aorta in view of almost equal blood pressure in both upper limbs. The results were satisfactory on short term follow up.

**Table:** 

Author's roles & responsibilities	
VA	Substantial contributions to the conception or design of the work; or the acquisition, analysis, or interpretation of data for the work; drafting the work or revising it critically for important intellectual content; agreement to be accountable for all aspects of the work in ensuring that questions related to the accuracy or integrity of any part of the work are appropriately investigated and resolved; final approval of the version to be published
AM	Substantial contributions to the conception; agreement to be accountable for all aspects of the work; final approval of the version to be published
IJ	Substantial contributions to the conception; agreement to be accountable for all aspects of the work; final approval of the version to be published
MS	Substantial contributions to the conception; agreement to be accountable for all aspects of the work; final approval of the version to be published
AM	Agreement to be accountable for all aspects of the work in ensuring that questions related to the accuracy or integrity of any part of the work are appropriately investigated and resolved; final approval of the version to be published
